# Evaluation of renal oxygen saturation using photoacoustic imaging for the early prediction of chronic renal function in a model of ischemia-induced acute kidney injury

**DOI:** 10.1371/journal.pone.0206461

**Published:** 2018-12-17

**Authors:** Kenichiro Okumura, Junichi Matsumoto, Yasunori Iwata, Kotaro Yoshida, Norihide Yoneda, Takahiro Ogi, Azusa Kitao, Kazuto Kozaka, Wataru Koda, Satoshi Kobayashi, Dai Inoue, Norihiko Sakai, Kengo Furuichi, Takashi Wada, Toshifumi Gabata

**Affiliations:** 1 Department of Radiology, Kanazawa University School of Medical Science, Kanazawa, Ishikawa, Japan; 2 Division of Nephrology, Kanazawa University Hospital, Kanazawa, Ishikawa, Japan; 3 Department of Nephrology and laboratory, Kanazawa University, Kanazawa, Ishikawa, Japan; Universidade de Sao Paulo, BRAZIL

## Abstract

**Purpose:**

To evaluate the utility of photoacoustic imaging in measuring changes in renal oxygen saturation after ischemia-induced acute kidney injury, and to compare these measurements with histological findings and serum levels of kidney function.

**Material and Methods:**

Acute kidney injury was induced by clamping the left renal pedicle in C57Bl/6 mice, with a 35-min ischemic period used to induce mild renal injury (14 mice) and a 50-min period for severe injury (13 mice). The oxygen saturation was measured before induction, and at 5 time-points over the first 48 h after induction, starting at 4 h after induction. Oxygen saturation, histological score, kidney volume, and the 24 h creatinine clearance rate and serum blood urea nitrogen were also measured on day 28. Between-group differences were evaluated using a Mann-Whitney *U*-test and Dunn’s multiple comparisons. The association between oxygen saturation and measured variables was evaluated using Spearman’s correlation. A receiver operator characteristic curve was constructed from oxygen saturation values at 24 h after heminephrectomy to predict chronic renal function.

**Results:**

The oxygen saturation was higher in the mild than severe renal injury group at 24 h after induction (73.7% and 66.9%, respectively, *P*<0.05). Between-group comparison on day 28 revealed a higher kidney volume (*P* = 0.007), lower tubular injury (*P*<0.001), lower serum level of blood urea nitrogen level (*P* = 0.016), and lower 24 h creatinine clearance rate (*P* = 0.042) in the mild compared with the severe injury group. The oxygen saturation at 24 h correlated with the 24 h creatinine clearance rate (*P* = 0.036) and serum blood urea nitrogen (*P*<0.001) on day 28, with an area under the receiver operating curve of 0.825.

**Conclusion:**

Oxygen saturation, measured by photoacoustic imaging at 24 h after acute kidney injury can predict the extent of subsequent histological alterations in the kidney early after injury.

## Introduction

Acute kidney injury (AKI) is characterized by the sudden loss of renal function [1, 2]. AKI frequently develops in seriously ill patients, from various causes, and commonly leads to a subsequent onset of chronic kidney disease, poor prognosis, and high risk of mortality [3–5]. Clinical diagnosis and staging of AKI are routinely based on serum creatinine and urinary output [1]; however, these parameters might not be reliable predictors of AKI prognosis.

Renal ischemia-reperfusion injury is an *in vivo* model of human AKI that leads to inflammation, decreased renal perfusion, loss of renal function, and progressive renal fibrosis [6–8]. Therefore, a decrease in renal perfusion after an ischemic event might be a critical pathophysiological factor of ischemia-induced AKI [7, 9]. As such, early change in renal microcirculation and oxygen supply after an ischemia-reperfusion event may be an important predictor of recovery from AKI, as well as of the transition from AKI to chronic kidney disease. However, the absence of noninvasive techniques for real-time imaging of these changes, combined with the lack of reliable physiological biomarkers of these changes, has limited the study of renal microcirculation as a predictor of renal function after AKI.

Recently, the Acute Dialysis Quality Initiative workgroup published a set of physiological biomarkers for the early identification and differential diagnosis of AKI [10]. As well, photoacoustic imaging (PAI) has emerged as a noninvasive technique to measure the change in renal oxygen saturation of hemoglobin (sO_2_), which could allow early detection of kidney injury. PAI is a functional optical imaging technique that uses thermoelastic expansion of tissues under illumination to generate contrast [11], with optical absorption at the excitation wavelength creating ultrasound waves that are detected by a transducer [11, 12]. In biological tissues, the major optical absorbers are hemoglobin, melanin, and water, which, therefore, serve as endogenous contrast agents for PAI [12, 13]. PAI is increasingly used to map tumor hypoxia and monitor changes in tumor oxygenation after treatment [14, 15]. However, very few studies have validated PAI results against those of standard radiological techniques [16, 17]. We hypothesized that renal sO_2_ mapping, measured using PAI, could serve as a noninvasive biomarker of the severity of AKI and thus predict the extent of subsequent histological alterations of the kidney early after injury. Therefore, the purpose of this study was to determine whether sO_2_ mapping does in fact show serial changes in the kidney following ischemia-induced AKI and to compare this mapping to histological examination and 24 h creatinine clearance (CCr) and serum blood urea nitrogen (BUN) level.

## Material and methods

### Animals

**Fig 1:**
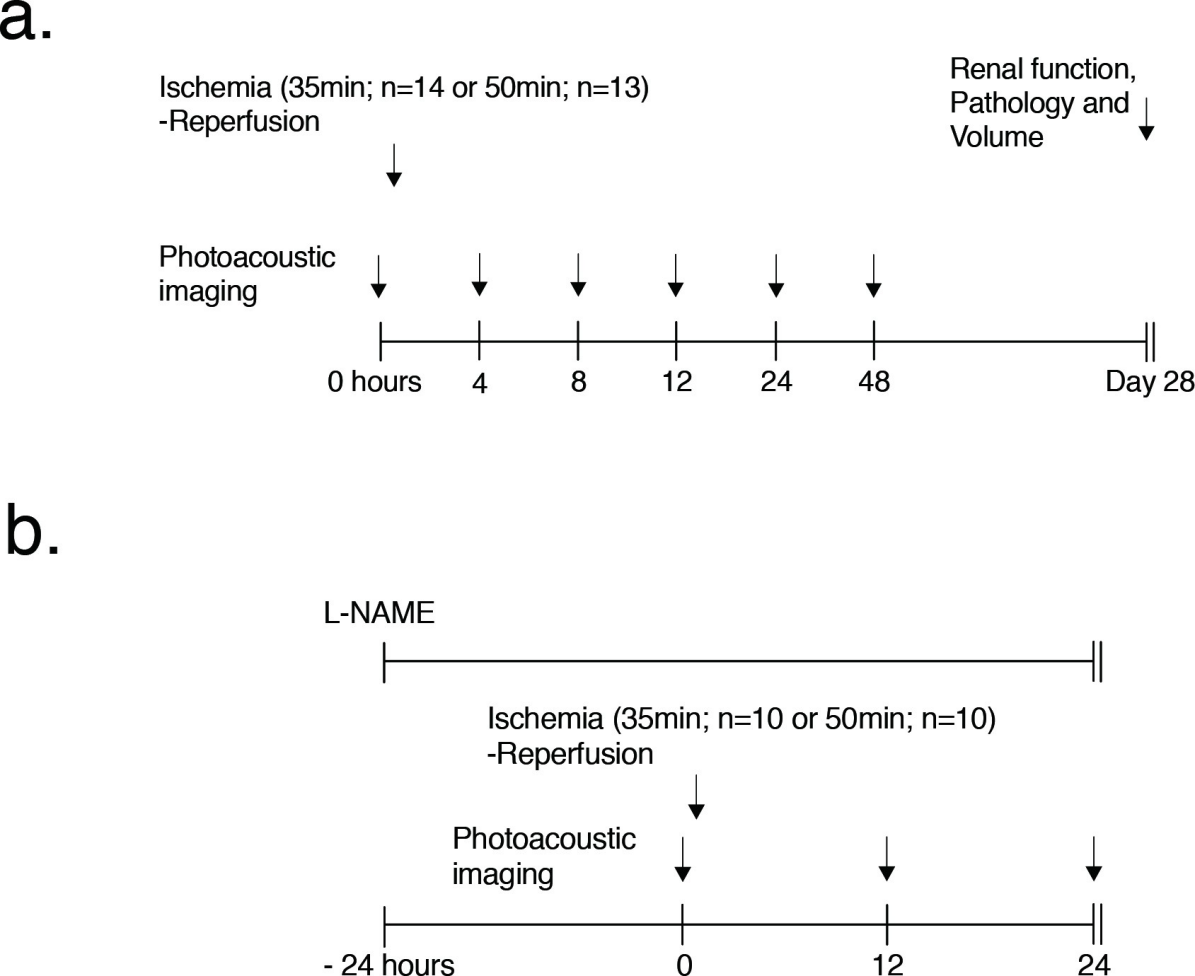
Experimental design in our renal ischemic-reperfusion model. (a) PAI was performed at different time points (before injury and at 4, 8, 12, 24 and 48 h after ischemia-reperfusion injury). The time period of renal ischemia-reperfusion was 35 min to induce mild AKI (14 mice) and 50 min to induce severe AKI (13 mice). Renal function, histological pathology and volume were measured 28 days after AKI induction. (b) L-NAME was administered from 24 h before ischemia-reperfusion to 24 h after reperfusion. PAI was performed at different time points (before injury and at 12 and 24 h after ischemia-reperfusion injury). The period of renal ischemia-reperfusion was 35 min to induce mild AKI (10 mice) and 50 min to induce severe AKI (10 mice).

The local animal protection committee approved the experimental protocol. Thirty-five male C57Bl/6N mice, 6–8 weeks old and weighing 20–25 g, were used: fifteen in mild and severe AKI groups and five in the control group. One of the fifteen (7%) mice in the mild AKI group and two of the fifteen (13%) mice in the severe ischemia group was excluded for the deterioration of the general condition. Thus, thirty-two mice were analyzed in total, with AKI induced in 27 and 5 serving as controls (Fig 1A). Additionally, to measure nitric oxide (NO) and hydrogen peroxide (H_2_O_2)_ at 12 h and 24 h after ischemia-reperfusion, respectively, eight mice each in the severe and mild AKI groups and five mice for control were used. To evaluate the effect of the NO synthase inhibitor (Nω-Nitro-L-arginine methyl ester hydrochloride; L-NAME; Sigma-Aldrich), another 24 mice were employed, eight each in the mild, severe AKI and control groups respectively (Fig 1B). All mice were euthanized by carbon dioxide insufflation after the experiments.

### Ultrasound (US) and photoacoustic imaging (PAI)

Imaging was performed using a Vevo LAZR small-animal US and PA imaging system (Visualsonics), with a LZ-550 linear-array transducer (256 elements, 40-MHz center frequency, 27-MHz bandwidth) used to acquire all images. The tunable laser supplied 10–20 mJ per pulse over the 680–970-nm wavelength range, with a pulse repetition frequency of 10 Hz. Once initialized, the system was switched to the oxy/hemo mode to measure sO_2_ using the following parameters: depth, 10.00 mm; width, 14.08 mm; wavelength, 750 and 850 nm for the total hemoglobin concentration threshold (Hbt), and sO_2_/Hbt, respectively. Renal sO_2_ mapping were pseudocolorized and superimposed on B-mode US images.

### Renal ischemia-reperfusion injury

AKI was induced by transient unilateral clamping of the left renal pedicle for a period of 35 min to induce mild AKI (14 mice) and 50 min for severe AKI (13 mice), as per a previously published method, ensuring low mortality [18, 19]. Another 5 mice were used as controls for comparison of renal function at 28 days. Under pentobarbital sedation (30 mg/kg, intraperitoneally), an incision was made on the left side of the back and the renal pedicle was bluntly dissected. A vascular clamp was applied to the renal pedicle for either 35 or 50 min. After removal of the clamp and closure of the incision, the mice were returned to their cages and monitored until fully awake. Animals received a standard diet with free access to tap water.

### Photoacoustic imaging

Mice in the AKI groups and controls underwent PAI examination continuously at six different time points: before injury (just after re-perfusion) and at 4, 8, 12, 24, and 48 h after ischemia-reperfusion. And mice administrated of L-NAME were also examined at 12 and 24 h after. For imaging, the mice were lightly anesthetized with isoflurane (2.0%) and positioned on a heated platform, with body temperature, heart rate, and respiration rate monitored. All images were acquired in the long axis by placing the probe directly over the back incision. Before sO_2_ measurement, B-mode and Doppler US images were acquired to evaluate renal blood flow status and identify the region of interest in the renal cortex, including the corticomedullar junction. The evaluation of AKI by PAI was performed by two radiologists with 10- and 7-years of experience, who were blinded to the identity of the animal groups. Determination of anatomical structures and ROI placements were performed with discussion.

### Determination of NO and hydrogen peroxide concentrations

The NO and H_2_O_2_ concentration in the kidney were measured using a QuantiChrom Nitric Oxide Assay Kit (BioAssay Systems, Hayward, CA, USA) and an OxiSelect Hydrogen Peroxide/Peroxidase Assay Kit (Cell Biolabs, San Diego, CA, USA), respectively, in accordance to the manufacturer’s protocol.

### Administration the nonselective NO synthase inhibitor *N*_*ω*_ -nitro-L-arginine methyl ester

Ischemia-reperfusion mice were administered L-NAME dissolved in drinking water (dose 100 mg/kg/d (1 g/l)). PAI was performed at 12 h and 24 h after ischemia-reperfusion (Fig 1B).

### Assessment of renal function using serum blood urea nitrogen (BUN) and the 24 h creatinine clearance rate (CCr)

To assess the renal function of the injured kidney, we performed contralateral nephrectomy 28 days after ischemia. Five to seven days after the heminephrectomy, serum and 24 h urine samples were collected. Serum creatinine, BUN and urine creatinine levels were determined using the Hitachi Clinical Analyzer 7180 (Hitachi Ltd., Tokyo). The creatinine clearance rate was calculated as follows: urine creatinine (mg/dL)/serum creatinine (mg/dL) × urine volume (mL)/collection period (min).

### Kidney volume and histopathological evaluation

The volume of the kidney was measured using B-mode US just after injury and at day 28, and the length of the long axis × the length of the short axis^2^ × 1/6 was calculated, with measurements obtained at the largest cross-section of the kidney. After that, the evaluation of acute tubular injury was performed by a nephropathologist with >15 years of experience, who was blinded to group allocation, using a previously described method [20]. Briefly, kidney tissue was subjected to periodic acid Schiff's staining for histological analysis. The tubular damage index was determined by assessing tubular necrosis, atrophy, and intra-tubular debris for 20 renal corticomedullary lesions in randomly selected microscopic fields (×200 magnification) in the kidney cross-section, and graded as follows: 0, none; 1, <20%; 2, 20–40%; 3, 40–60%; 4, 50–80%; and 5, >80% of the field. The tissue was also subjected to AZAN staining to assess interstitial fibrosis. In each case 5–7 randomly chosen microscopic fields from the renal cortico-medullary junction were captured. For digital analysis, ImageJ software (Rasband W, National Institutes of Health, USA) and a macro code, which can be tailored using with hue, saturation, brightness color filtering, were used.

### Statistical analysis

Statistical analyses were performed using GraphPad software (version 7.00 for Mac; GraphPad Software, San Diego, USA). The Mann-Whitney *U*-test was used to evaluate the differences in histopathological examination results, NO and H_2_O_2_ concentrations at 12 and 24 h, relative renal volumes, serum BUN levels, and 24 h CCr on day 28 between the mild and severe AKI groups. Longitudinal changes in renal sO_2_ over the first 48 h after injury were evaluated using an analysis of variance for repeated measurements followed by Dunn’s multiple comparison post-hoc test, with *P* values adjusted for multiple comparisons. The correlation between renal sO_2_ and serum BUN and the 24-h CCr at day 28 was evaluated using linear regression and Spearman’s coefficient of correlation. The sO2, serum BUN, 24-h CCr, pathological score, and kidney volume were described by their median value and range. *P*-values <0.05 were considered significant. A receiver operator characteristic (ROC) curve was constructed by sorting all kidneys in both the mild and severe AKI groups by their sO_2_, and the true-positive and false-positive rates for identifying severe AKI were calculated using increasing sO_2_ at 24 h for step-wise classification, with the serum BUN level, 24 h CCr, and relative renal volume at day 28 used as the ground truth.

## Results

### Change in cortical renal oxygen saturation

**Fig 2:**
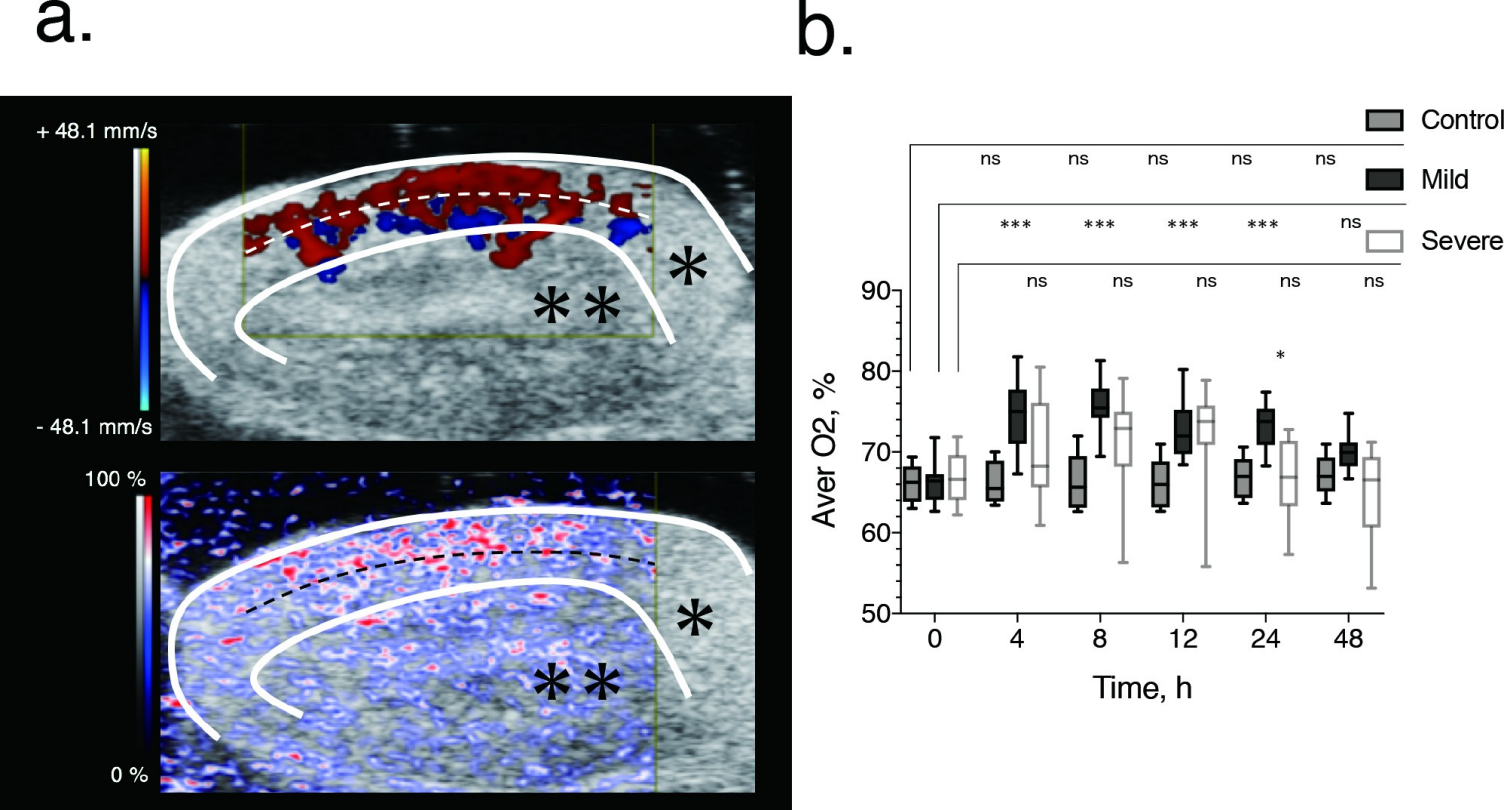
Image-based quantification and longitudinal time course of oxygen saturation after renal ischemia-reperfusion in the mild and severe ischemia groups. (a) Normal renal blood flow (top) and renal tissue saturation (bottom), showing blood flow in the cortex, around the medullary border region, indicative of high oxygen saturation. The white and black dashed lines indicate the cortical medullary border. The regions in which Doppler ultrasound signals of the renal arcuate arteries and veins are observed correspond to the medulla and external cortex. The white line indicates the kidney parenchyma. The color map (graduated from red to blue), which is based on the principle of using the difference in light absorption wavelength between oxygenated hemoglobin (850 nm) and deoxygenated hemoglobin (750 nm) as a signal, shows the change from high (red) to low (blue) oxygen saturation. The white lines delimit the renal parenchyma (*) and the area outside represents the urinary tract (renal pelvis) (**). Scale bar, 2 mm. (b) Box-and-whisker plots of the time course of renal oxygen saturation for the mild (black) and severe (red) ischemia-reperfusion group. The box indicates the 25^th^ and 75^th^ quartiles, with the line inside each box indicating the median and the whiskers, the maximum and minimum renal oxygen saturation. The asterisk indicates significant between-group differences (*P*<0.050) in renal oxygen saturation at 24 h after AKI-induction using Dunn’s multiple comparisons as a non-parametric test. Comparisons between oxygen saturation before injury and at the different time points of measurement after injury (4, 8, 12, 24 and 48 hours) were also evaluated using the Dunn’s multiple comparisons. *p < 0.050, ***p < 0.001, ns not significant.

Using a high-frequency probe for B mode and Doppler imaging, the renal cortex was clearly discriminated from the medulla, with successful identification of the region of interest and placement of the probe for data acquisition in all mice. In normal state, sO_2_ was higher in the cortex than in the medulla (Fig 2A). The main results of PAI analysis are shown in Fig 2B.

Serial analysis of sO_2_ at each time point revealed a significantly higher sO_2_ in the mild AKI group (median, 73.7%; range, 68.3%-77.4%) than in the severe AKI group (median, 66.9%; range, 57.3%-72.8%) at 24 h (*P*<0.05), despite the cortical perfusion in Doppler images being almost identical for the two groups. There was no significant difference between the severe and mild AKI groups in terms of the median sO_2_ level in the pre-ischemic state (median, 66.3% and 66.4%, respectively).

Serial PAI results are shown in Fig 2B. In the mild AKI group, the median sO_2_ level was higher than at baseline (median, 66.4%) at 4 (median 75.0%; *P* < 0.001), 8 (median, 75.5%; *P* < 0.001), 12 (median, 72.0%; *P* < 0.001), and 24 h (median, 73.8%; *P* < 0.001) after ischemia. The increase in sO_2_ peaked at 8 h, gradually returning to baseline level thereafter. The median sO_2_ level also showed a slight increase from baseline (median, 69.1%) at 4 (73.0%) and 12 h (73.8%) after ischemia in the severe AKI group, although these changes were not significant. In the control group, the median sO2 remained constant at baseline (median, 66.2%) and at 4 (65.5%), 8 (65.6%), 12 (66.0%), 24 (67.0%) and 48 h (67.0%).

### Change in renal tissue NO and oxidative stress marker (H_2_O_2_) levels

**Fig 3:**
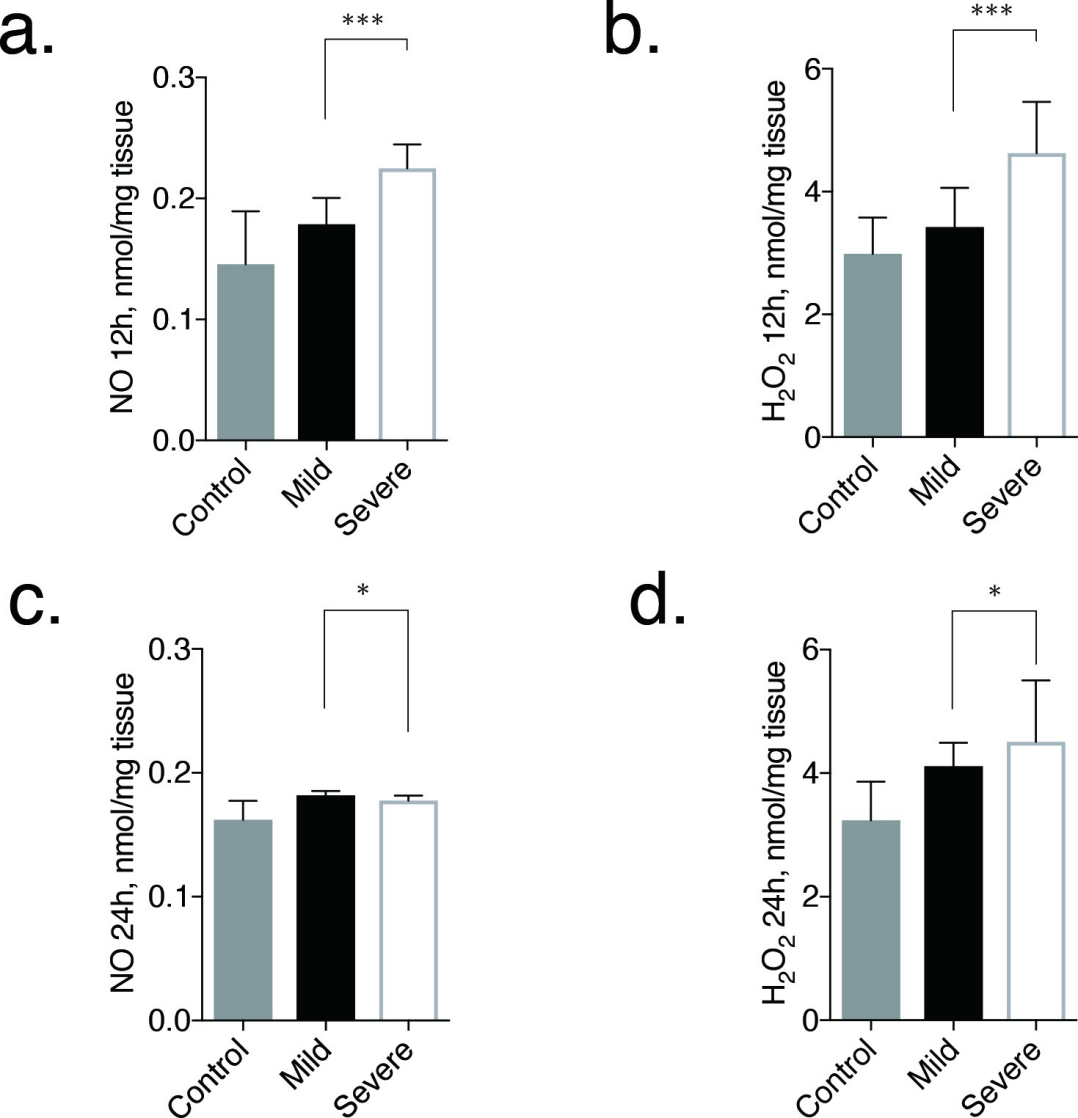
Evaluation of NO and H_2_O_2_ concentrations in kidneys at 12 and 24 h after ischemia-reperfusion injury. The bar graphs show the grade of renal tubule injury and fibrosis at day 28, determined from pathological tissue specimens. Error bars represent the range of the median. There is a statistical difference in NO and H_2_O_2_ concentrations in the kidney at 12 h (*P* < 0.001) and 24 h (*P* < 0.050) between the mild and severe AKI groups. *p < 0.050, ***p < 0.001.

The main results of NO and H_2_O_2_ analysis are shown in Fig 3.

At 12 h after ischemia-reperfusion, the NO concentrations were significantly higher in the severe AKI group (median, 0.255 nmol/mg tissue; range, 0.199 nmol/mg tissue -0.245 nmol/mg tissue) than in the mild AKI group (median, 0.179 nmol/mg tissue; range, 0.164 nmol/mg tissue -0.201 nmol/mg tissue; *P* = 0.0003; Fig 3A).

The same trend was also observed in H_2_O_2_ between severe (median, 4.63 nmol/mg tissue; range, 4.35 nmol/mg tissue -5.46 nmol/mg tissue) and mild (median, 3.42 nmol/mg tissue; range, 3.07 nmol/mg tissue -4.06 nmol/mg tissue; *P* = 0.0002; Fig 3B) AKI groups.

On the other hand, at 24 h after ischemia-reperfusion, NO concentration showed significantly higher value in the mild AKI group (median, 0.182 nmol/mg tissue; range, 0.177 nmol/mg tissue -0.185 nmol/mg tissue) than in severe AKI group (median, 0.178 nmol/mg tissue; range, 0.166 nmol/mg tissue -0.182 nmol/mg tissue; *P* = 0.035; Fig 3C). Nevertheless, H_2_O_2_ concentration stayed higher in the severe AKI group (median, 4.51 nmol/mg tissue; range, 4.19 nmol/mg tissue -5.51 nmol/mg tissue) than in mild AKI group (median, 4.11 nmol/mg tissue; range, 3.84 nmol/mg tissue −4.49 nmol/mg tissue; *P* = 0.015; Fig 3D) even at 24 h. In the control group, the median NO concentration was almost the same at 12 h (median, 0.146 nmol/mg tissue) and 24 h (0.162 nmol/mg tissue). The H_2_O_2_ concentration was also the same at 12 h (median, 2.99 nmol/mg tissue) and 24 h (3.24 nmol/mg tissue).

### Change in renal oxygen saturation upon administration of the nonselective NO synthase inhibitor *N*_*ω*_-nitro-L-arginine methyl ester

**Fig 4:**
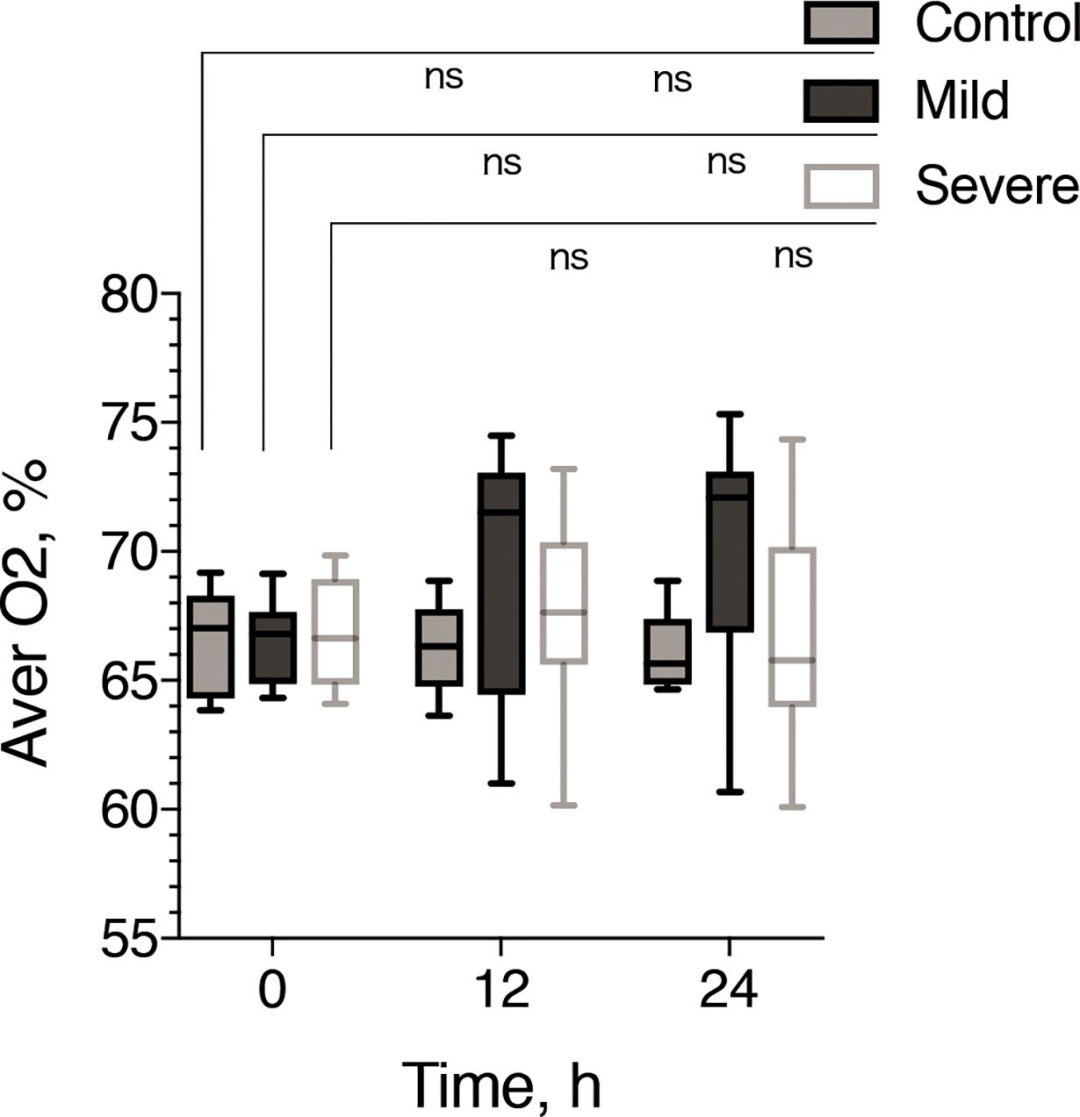
Longitudinal time course of oxygen saturation after renal ischemia-reperfusion in the mild and severe ischemia groups with L-NAME administration. Box and whisker plots of the time course of renal oxygen saturation for the mild (black) and severe (white) ischemia-reperfusion groups. The box indicates the 25^th^ and 75^th^ quartiles, with the line inside each box indicating the median, and the whiskers indicate the maximum and minimum renal oxygen saturations. The oxygen saturation before injury and at the different time points of measurement after injury (12 and 24 h) were also evaluated using Dunn’s multiple comparisons. ns, not significant.

In L-NAME administration series, the median sO_2_ at 12 and 24 h in the mild group (12 h: median, 71.51%; range, 61.01%-74.48%; 24 h: median, 72.08%; range, 60.68%-75.33%; Fig 4) demonstrated lower value than those in mild AKI group without L-NAME (Fig 2B), on the other hand there was no dramatic change in sO_2_ in severe AKI group (12 h: median, 67.63%; range, 60.15%-73.19%; 24 h: median, 65.79%; range, 60.09%-74.35%; Fig 4) between with and without L-NAME administration (Fig 2B). As a result, sO2 at 12 and 24 h in mild AKI group with L-NAME administration showed similar values with severe AKI group (with or without L-NAME administration) (Figs 2B and 4) and no statistically significant differences were observed between mild, severe AKI and control groups (0 h: median, 67.0%; 12 h: 66.3%; 24h: 65.6%) with L-NAME (Fig 4).

### Kidney volume and histological change

**Fig 5:**
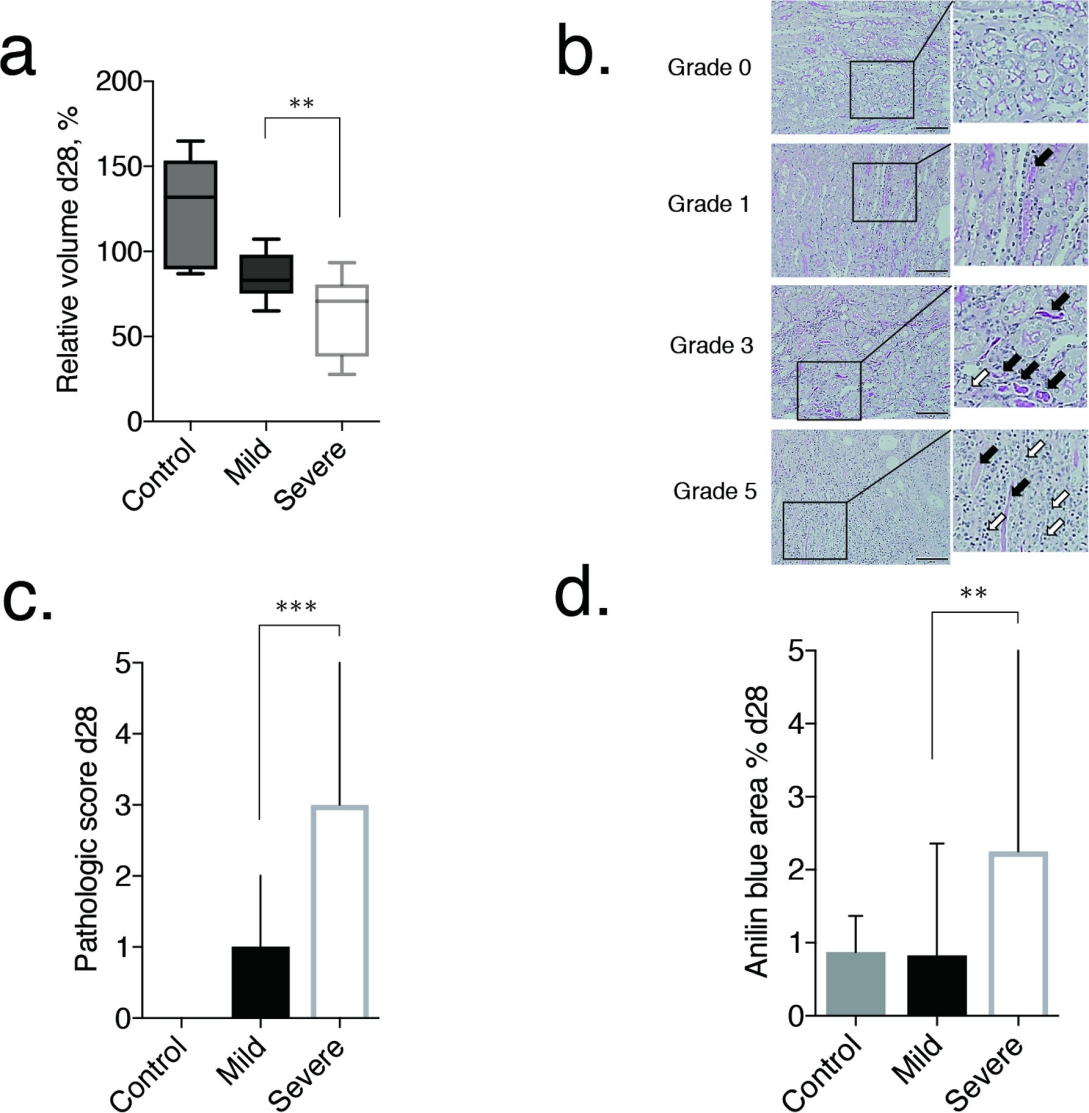
Volume and pathological evaluation of renal tubule injury in the chronic phase. (a) Box-and-whisker plot showing the relative volume at day 28. Each box in the plot represents the 25^th^ and 75^th^ quartiles, with the line inside each box indicating the median, and the whiskers, the maximum and minimum volume. The kidney volume was smaller at 28 days after AKI-induction in the severe (13 mice) than mild (14 mice) AKI group (*P* = 0.007). (b) Representative images of the grading of renal tubule injury (grades 0, 1, 3, and 5). In grade 0, tubules with normal brush border were seen. Some casts were detected in grade 1, and the number of casts increased in grade 3. In grade 5, more tubular atrophy, loss of tubules, and massive infiltration of leucocytes were observed (black arrows denote casts and white arrows denote infiltrating leucocytes). Original magnification, ×200. (c, d) Bar graphs showing the grade of renal tubule injury and fibrosis at day 28, determined from pathological tissue specimens. Error bars represent the range of the median. There is a significant difference in tubule damage (*P* < 0.001) and fibrosis (*P* < 0.010) in the kidney at day 28 between the mild and severe groups. **p < 0.010, ***p < 0.001.

The volume of the ischemic kidney, measured 28 days after the ischemia-reperfusion surgery, decreased from baseline in all mice in the severe AKI group, with a volume loss >40% identified in 9 of the 13 mice in this group (69.2%). By comparison, in the mild AKI group, the kidney volume remained close to the baseline volume in all mice. Therefore, the volume values, relative to baseline, were significantly lower in the severe AKI group (median, 70.8%; range, 27.8%-93.4%) than in the mild AKI group (median, 83.1%; range, 65.1%-107.3%; *P* = 0.007; Fig 5A). In the mild and severe group, the values were lower than those of the control (median, 131.9%; range, 88.9%-164.9%).

Histopathological abnormalities were observed both in mild and severe AKI groups at 28 days after injury, however, the severities were different between two groups. Although early atrophy of single nephrons in the cortex, and perivascular and interstitial inflammatory cell infiltration were diffusely observed in both groups, these histological changes were more remarkable in the severe than in the mild AKI group. Adding to these atrophic changes of the renal cortex and interstitial inflammations, renal tubules were also damaged to various degrees in both groups (Fig 5B). These tubular damages were frequently observed in the corticomedullar junction area, observed as casts and loss of brush borders of the tubular epithelium. Although there was almost no tubular damage in the controls (median, 0; range, 0–0), tubular damage were significantly more pronounced in the severe AKI group compared with the mild AKI group (median, 3; range, 0–5 versus median, 1; range, 0–2, respectively, *P*<0.001; Fig 5C). In the control group, the score was 0. Fibrosis in the corticomedullar junction area was significantly more pronounced in the severe AKI group than in the mild AKI group (median, 2.252%; range, 0.199%-18.959% versus median, 0.827%; range 0.076%-2.361%, respectively, *P*<0.010; Fig 5D). The degree of fibrosis in mild group was as mild as that in controls (median, 0.874%; range, 0.393%-1.370%).

### Renal function

**Fig 6:**
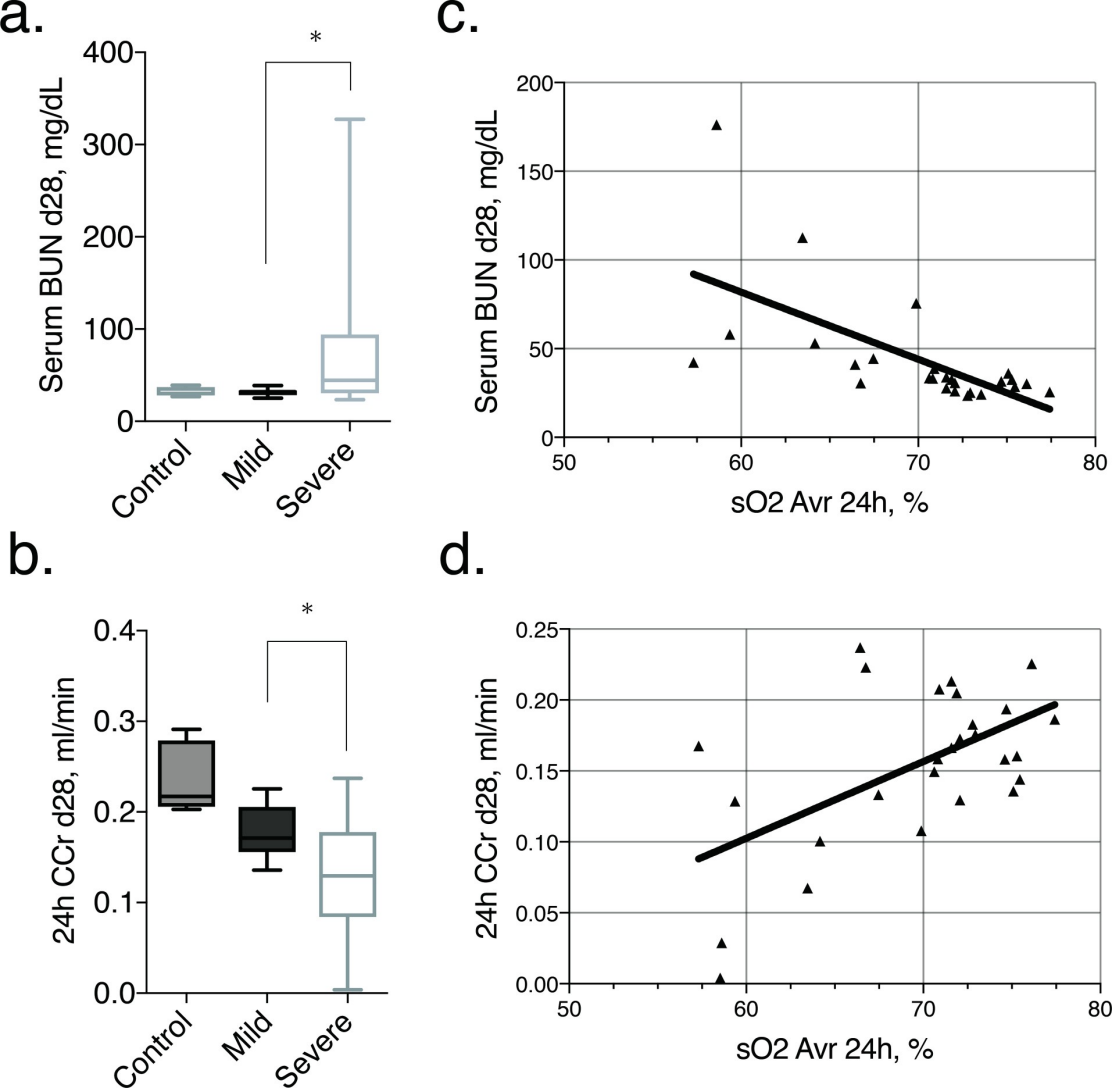
Relationship between renal function in the chronic phase and acute stage renal oxygen saturation. (a, b) Box-and-whisker plots show serum BUN and 24 h creatinine clearance for the control (5 mice) group and the mild (14 mice) and severe (13 mice) AKI groups. Each box in the plot represents the 25^th^ and 75^th^ quartiles, with the line inside each box indicating the median, and the whiskers, the maximum and minimum serum BUN and 24 h creatinine clearance. There is a statistically significant difference (*P* = 0.016 and 0.042, respectively) between the mild and severe AKI groups at day 28, using the non-parametric Mann-Whitney U test. (c, d) Scatterplots show the correlation between renal oxygen saturation at 24 h and kidney function at day 28, with this correlation being significant for both serum BUN (*r* = -0.736, *P*<0.001) and the 24-h creatinine clearance (*r* = 0.405, *P* = 0.036). *p < 0.050.

At day 28, the serum BUN value in the mild AKI group (median, 32.1 mg/dL; range, 25.2 mg/dL-38.8 mg/dL) was almost the same as that for the control group. In contrast, serum BUN level was significantly higher in the severe AKI group (median, 44.4 mg/dL; range, 23.6 mg/dL-327.6 mg/dL) than in the control group (*P* = 0.016; Fig 6A). The 24-h CCr values at 28 days after ischemia were lower in both AKI groups than in the control group (median, 0.217 ml/min; range, 0.203 ml/min-0.291 ml/min). Moreover, the 24-h CCr was significantly lower in the severe AKI group (median, 0.130 ml/min; range, 0.004 ml/min-0.240 ml/min) than the mild AKI group (median, 0.170 ml/min; range, 0.140 ml/min-0.230 ml/min; *P* = 0.042; Fig 6B).

The correlation between sO_2_ at 24 h after AKI and the serum BUN and 24-h CCr is shown in Fig 6. Interestingly, the sO_2_ at 24 h was negatively correlated with the serum BUN level (*r* = - 0.736, *P*<0.001; Fig 6C) and positively correlated with the 24-h CCr (*r* = 0.405, *P* = 0.036; Fig 6D) at day 28.

**Fig 7:**
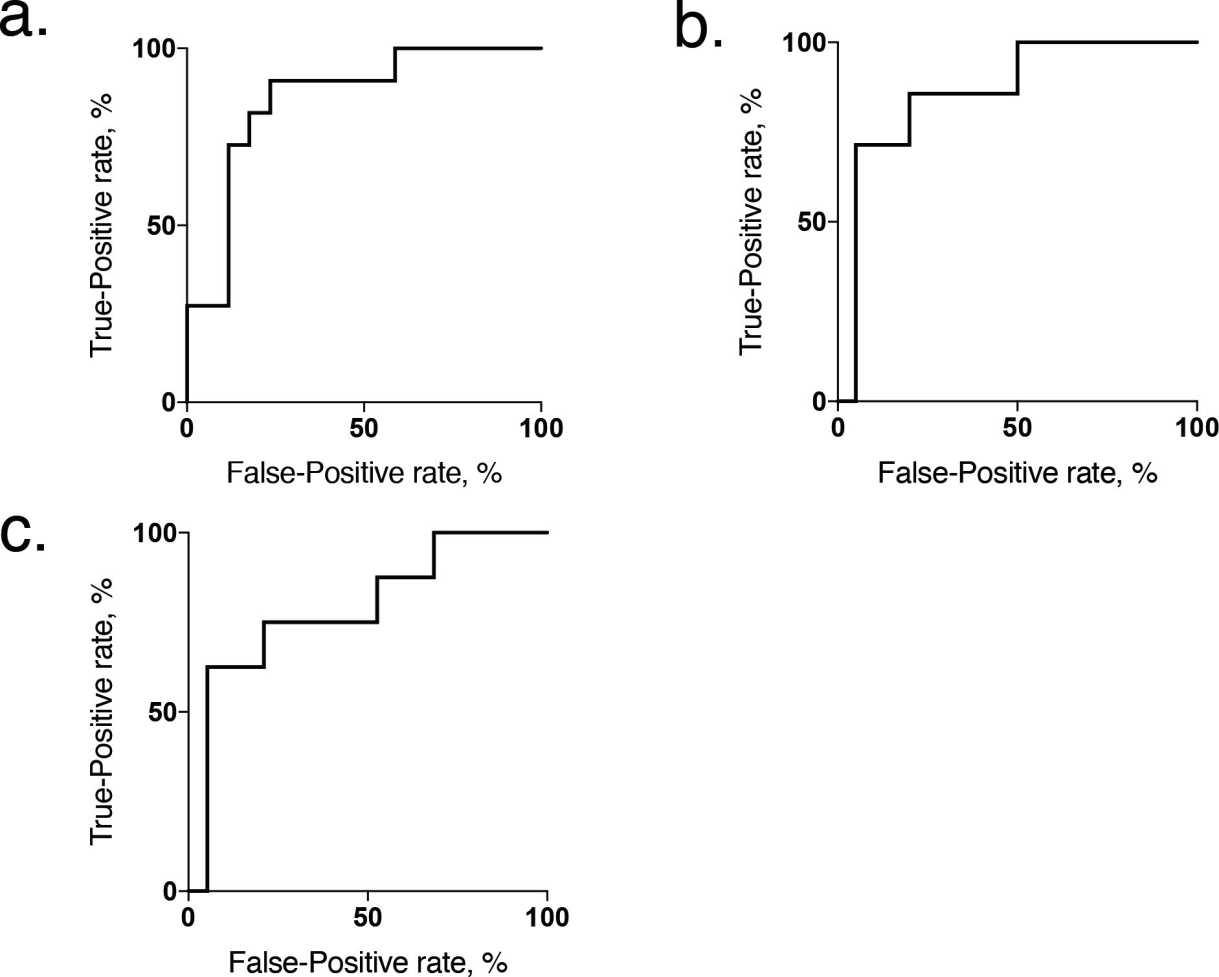
Receiver operator characteristic curve showing renal oxygen saturation at 24 h. (a) Receiver operator characteristic curve showing renal oxygen saturation at 24 h acting as a moderate classifier of chronic renal function, measured by the serum BUN at day 28. The optimal sO2 cutoff value at 24 h to differentiate better from worse renal failure at day 28 was 71.59%, with an area under the curve is 0.825 (*P* = 0.008). (b) Measured in terms of the 24 h CCr at day 28, the optimal sO2 cutoff value at 24 h to differentiate mild from severe renal failure was 65.3%, with an area under the curve of 0.864 (*P* = 0.005). (c) Measured in terms of the relative renal volume, the optimal sO2 cutoff value at 24 h to differentiate mild from severe renal atrophy was 65.3%, with an area under the curve of 0.790 (*P* = 0.020).

The diagnostic value of sO_2_ at 24 h after ischemia for predicting a decrease in renal function, defined as a BUN value greater than the value of serum BUN in control animals (median, 30.0 mg/dL; range, 26.8 mg/dL-39.0 mg/dL), was further examined, with this value being used as the ground truth to define either better or worse renal function. On ROC analysis, sO_2_ at 24 h, with a cut off value of 71.59%, discriminated better renal function from worse with a sensitivity of 100% and specificity of 70%, with an area under the curve of 0.825 (Fig 7A).

The 24 h CCr and relative renal volume values in the control animals were greater than the values in the mild and severe ischemia groups. Thus, the diagnostic value of sO_2_ at 24 h after ischemia for predicting a decrease in the renal function, defined as values greater than the median values of 24 h CCr and relative renal volume in severe ischemia animals (0.130 ml/min and 70.8%, respectively), was further examined using this value as the ground truth to define either mild or severe renal dysfunction. On ROC analysis regarding 24 h CCr, sO_2_ at 24 h, with a cut off value of 65.3%, discriminated with a sensitivity of 71% and specificity of 95%, with an area under the curve of 0.864 (Fig 7B). Also, depended on relative renal volume, sO_2_ at 24 h which cut off value was 65.3% discriminated severe renal failure from mild failure at day 28 with a sensitivity of 62.5% and specificity of 94.7%, with an area under the curve of 0.790 (Fig 7C).

## Discussion

Our serial analysis of renal sO_2_ using PAI in an ischemia-reperfusion model revealed the following features of AKI. Over the first 4–48 h after induction, renal sO_2_ was higher in the mild than severe AKI group, this difference reaching a maximum at 24 h post-ischemia. The absolute sO_2_ value at 24 h was predictive of renal function at 28 days (AUC 0.825). It is important to note that we used an established model of ischemia-reperfusion in C57Bl/6N mice [18]. In addition, the extent of renal pathology and kidney volume between the mild and severe AKI groups at 28 days were comparable to previously reported values [21]. Therefore, we deem that our ischemia-reperfusion procedure was performed properly.

Regardless of the underlying cause, AKI is caused by a transient decrease in renal microcirculation and oxygenation, with the resulting hypoxia triggering an inflammation that leads to irreversible fibrosis and tubular damage of renal tissue [22]. Although the detailed mechanism remains unknown, tissue hypoxia is considered as an essential factor of AKI [23]. Although clinical benefits of drug therapy in preventing the progression of AKI to chronic renal failure have been reported in clinical trials, the prognosis of patients with AKI remains poor [4]. The difficulty with early detection and the lack of reliable biomarkers to evaluate the severity of AKI contribute to this poor prognosis. Currently, AKI diagnosis is based on the glomerular filtration rate (GFR), estimated from creatinine values in excreted urine. Because of the significant delay between the onset of AKI and change in GFR, the GFR does not serve as a sensitive marker of AKI [24]. Functional magnetic resonance imaging (Blood oxygen level dependent magnetic resonance imaging; BOLD-MRI) has been used to evaluate intrarenal oxygenation under physiological and pathophysiological conditions [25]. Pohlmann et al. reported a strong correlation between the BOLD MRI signal and the partial pressure of oxygen (pO2) *in vivo* [26]. Hueper et al. reported on the utility of arterial spine labelling (ASL) in describing renal perfusion in C57Bl/6N mice, which predicted chronic renal function from measures obtained as early as day 7 after ischemia [21]. In our study, we demonstrate the utility of PAI in improving this timeline by providing a separate measure of the tissue concentration of hemoglobin (HbO_2_) and deoxyhemoglobin (Hb), as well as directly quantifying tissue oxygen saturation [27, 28]. Specifically, we demonstrate that renal sO_2_, measured at 24 h after onset of renal ischemia, can reliably predict renal function in the late phase (28 days) at a high rate (AUC 0.825). This is an early biomarker for AKI to estimate late phase renal function. The use of PAI for *in vivo* sO_2_ measurements in experimental models has previously been reported [16, 29]. Moreover, Rich et al. reported a good correlation between PAI and BOLD-MRI [30]. Because of its reliability in measuring sO_2_ *in vivo*, PAI is currently being developed for clinical use for the detection of breast, prostate, and gallbladder cancer [31–33].

The underlying mechanism leading to a difference in renal sO_2_ between severe and mild AKI mice is not clear. Renal oxygenation depends on a balance between microvascular blood flow and oxygen consumption with the produced NO that could finally result in the microvascular injury and the loss of renal function. A recent study also demonstrated an increase in oxygen return from the renal vein in the kidney in a rat model after reperfusion [34]. Furthermore, overproduction of H_2_O_2_, which is another oxidative stress marker that works independently from NO has been reported [35]. In our study, one possible explanation on the increase in renal sO_2_ at 12 h after ischemia-reperfusion might be due to an increase of tissue NO. Additionally, the remarkable decrease of tissue NO concentration observed in severe AKI group presumably the main reason for lower sO_2_ at 24 h after injury. The effect of tissue H_2_O_2_ on sO2 remained unclear, however, constant higher value of H_2_O_2_ in severe AKI group could be considered as the important factor that progressed tissue damage [35].

Persistent vasoconstriction and blood flow reduction could be an important factor, triggering a vascular response leading to endothelial cell damage at microvascular level [36]. Another study demonstrated a decrease in microvascular oxygenation of the renal cortex despite normal systemic hemodynamics and O_2_ delivery in renal of pigs following reperfusion after 45 min of ischemia [37]. Although these animal species are different from the mice model we used, these findings do support the occurrence of a change in oxygen saturation and renal blood flow after ischemia-reperfusion. We propose that fine changes in renal blood flow and oxygen consumption induced by the NO between the mild and severe AKI groups resulted in the greater tubule injury in the severe group, which influenced renal blood flow and kidney volume, resulting in the between-group difference in sO_2_ that we measured using PAI during the acute phase after reperfusion.

The limitations of our study include the small number of animals in each group and the diversity of kidney function among animals within each group. To minimize error, we also placed the probe for PAI over the incision performed on the back for AKI-induction, rather than using PAI completely as a noninvasive imaging technique. Therefore, future work is needed for the use of PAI to measure sO_2_ transcutaneously. Additionally, pathological background at 24 h that could be a clue for explanation of the difference in sO_2_ at 24 h was not examined.

In conclusion, PAI provided a sensitive measure of the change in sO_2_ in the acute phase of AKI, with the sO_2_ at 24 h after AKI being predictive of renal function in the chronic phase. Thus, sO_2_ mapping may be very valuable for clinical follow-up of patients at risk for AKI and for studies using experimental models of renal disease.
